# First Documented Case of Complete Leucism in the Long-Eared Owl (*Asio otus*) in Bulgaria

**DOI:** 10.3390/biology15191777

**Published:** 2026-10-07

**Authors:** Rusko Petrov, Simona Krasteva

**Affiliations:** 1 Department of General Animal Husbandry, Faculty of Veterinary Medicine, Trakia University, 6000 Stara Zagora, Bulgaria; rusko.petrov@trakia-uni.bg; 2 Green Balkans—Stara Zagora NGO, Boruygrad Street 30, 6000 Stara Zagora, Bulgaria; 3 Faculty of Veterinary Medicine, Trakia University, Student Campus, 6000 Stara Zagora, Bulgaria

**Keywords:** leucism, pigmentary anomalies, *Asio otus*, Long-eared owl, Strigiformes, wild birds, Bulgaria

## Abstract

Leucism is a hereditary anomaly affecting the coloration of avian species. It is defined by partial or complete loss of plumage coloration. Unlike albinism, which results in ocular depigmentation, leucistic birds have normal iris color. Pigment abnormalities within wild bird populations are rare. In the order Strigiformes, such cases have mainly been recorded as individual reports. This research introduces the first documented case of complete leucism in the Long-eared owl (*Asio otus*) in Bulgaria. A retrospective analysis of clinical records from the Wildlife Rescue Center (“Green Balkans”, Stara Zagora) between 1995 and 2026 revealed that, among 1098 admitted individuals, only one exhibited this phenotype. In February 2016, a leucistic individual with multiple fractures was admitted into the center. The bird displayed the hallmark phenotypic features of complete leucism. Following treatment and rehabilitation, the owl regained full flight capacity and was successfully released into the wild in June 2016. This case confirms the extreme rarity of leucism in the Long-eared owl and highlights the essential function of long-term rescue center databases in documenting rare morphological anomalies and advancing conservation research.

## Simple Summary

In wild birds, color anomalies are extremely rare; as such, they offer significant understanding into wildlife genetics and survival mechanisms. This study documents the first official record of a Long-eared owl with complete leucism in Bulgaria. Complete leucism is a genetic condition that causes an animal’s feathers, beak, and feet to appear white, while its eyes retain their normal color, which distinguishes it from albinism. By reviewing medical records from a national wildlife rescue center spanning thirty-one years, we found that out of 1098 admitted Long-eared owls, only one displayed this unique white color pattern—an admission frequency of less than 0.1 percent. The owl was treated for severe wing fractures, underwent rehabilitation, and was subsequently successfully released back into the wild. This result could show that even birds with color anomalies can still survive in nature when given proper medical care. In the long run, this work demonstrates the crucial value of wildlife rescue centers in recording rare genetic traits and how to evaluate the data accurately. Consequently, it supports biodiversity research while enabling better protection of native bird species.

## 1. Introduction

### 1.1. Background and Context

Plumage colour is an important adaptive trait that contributes to camouflage, communication, thermoregulation, sexual selection, and predator avoidance [1]. In wild populations, aberrations from normal pigmentation are quite rare [2] and have therefore attracted considerable interest from both genetic and ecological perspectives [3].

Leucism is one of the most frequently recorded cases of chromatic aberration in birds, due to their higher survival rate than albinos. This is partly because albino individuals lack melanin in the iris and retina, which can lead to difficulty with depth perception and also higher light sensitivity [4]. Another reason could be that albino individuals are even easier prey, due to their more noticeable nature [5]. Leucism is characterized by total or partial absence of functional melanocytes in the integument. It is a hereditary condition, resulting from a congenital anomaly in the differentiation, migration, or survival of melanoblasts, which are derived from the neural crest during embryonic development [1,2,6]. In contrast to albinism, which involves a complete biochemical failure of melanogenesis across all tissues due to an inherited lack of functional enzyme tyrosinase, leucistic birds retain normal ocular pigmentation [4,6,7]. This fundamental distinction occurs because ocular pigment cells originate from the neuroectoderm rather than the neural crest [1,4]. While partial leucism results in localized white patches, complete leucism involves a total absence of pigment across the entire plumage and extends to the bare integumentary structures, such as the rhamphotheca (outer beak covering) and podotheca (outer covering of the feet) [4,6,7].

Furthermore, complete leucism is distinct from other chromatic aberrations that produce pale or white phenotypes in birds [4,6,7]. Progressive greying shows a gradual, age-dependent loss of pigment over consecutive molts. Other chromatic aberrations such as dilution and ‘brown’ represent quantitative or qualitative reductions in melanin, producing washed-out greyish or pale brownish hues rather than pure white; and ‘ino’ causes strongly bleached plumage accompanied by lightened, reddish irides [4,6,8]. In the current documented Long-eared owl (*Asio otus*) specimen, the presence of snow-white plumage across the entire body, complete depigmentation of the rhamphotheca and podotheca, and fully pigmented orange-brown irides excludes these alternative conditions, identifying the phenotype as complete leucism [4,6,7]. However, because no genetic or histological analyses were performed, the diagnosis should be regarded as phenotype-based.

The order Strigiformes has over 200 species of nocturnal raptors [9]. The Long-eared owl ranges widely across the Holarctic region (Palearctic and Nearctic) [10] and is among the most widespread species of the order in Bulgaria, with an estimated breeding population of 3000 to 5000 pairs [11]. The species prefers mosaic landscapes that combine forests and open areas [12]. For reproduction, they typically use abandoned corvid nests. The typical plumage of the Long-eared owl consists of brownish-ochre hues with dark longitudinal streaks, providing effective camouflage. It is important to note that the characteristic auricular tufts in owls function as visual signals for communication and concealment, rather than for auditory reception [13]. In leucistic individuals, the depigmentation of these tufts may disrupt their protective pattern. Potentially reducing their functional capacity for camouflage, intraspecific signaling, or even mate choice [7,13].

### 1.2. Study Aim and Scope

There are very few documented cases of pigmentary anomalies in strigids, with observations largely limited to individual instances [14]. To the present day, no scientific record of leucism in the Long-eared owl has been published. The aim of this study is to report the first case ever observed of complete leucism in the Long-eared owl in Bulgaria and assess its admission frequency through a retrospective analysis of the center’s archives.

## 2. Materials and Methods

We present a rare case of complete leucism in a Long-eared owl. This chromatic aberration is the first documented record of the species in Bulgaria. The individual was admitted to the Wildlife Rehabilitation and Breeding Center of the “Green Balkans” NGO, Stara Zagora, Bulgaria, in February 2016. Data were obtained from a retrospective review of the center’s clinical records covering the 31-year period from January 1995 to August 2026.

## 3. Case Study

This study uses a retrospective examination of the institutional records maintained at the Wildlife Rehabilitation and Breeding Center of the “Green Balkans” Federation in Stara Zagora, Bulgaria (42°25′ N, 25°38′ E). Founded in 1992, the center functions as a specialized facility for the intake, veterinary care, treatment, rehabilitation, and release of injured, sick, and orphaned wild animals. The present study examines data from clinical records for all Long-eared owls admitted over a 31-year period, from 1 January 1995 to 30 August 2026. Using consistent record-keeping protocols across all years, for each owl entered into the records, the admission details were assessed, including the date of intake, the place where the owl was found, and the main reason for admission. As well as its clinical condition and whether or not any morphological abnormalities were present. Also, in order to identify any other cases of plumage pigmentation anomalies, the available medical records and photographic documentation were examined.

Over the course of the study, 1098 unique Long-eared owls were brought to the center, one of which showed a pigmentation abnormality, which gives an admission frequency of 0.091% (1/1098). This value represents the frequency of leucism in the center’s admission records and should not be interpreted as a population-level prevalence estimate. In addition, we compared our findings with data from the national monitoring of the Bulgarian breeding population—estimated at 3000 to 5000 breeding pairs [11]—and no similar pigmentary abnormalities have been recorded. This result points to the scientific importance of the current clinical case and confirms how rare the present phenotype is. The species identification was made using the standard morphological diagnostic criteria for bird species in Europe. The annual distribution of Long-eared owl admissions over the study period is summarized in Table 1. The data show a stable presence of admitted owls, with a distinct increase in intake after 2004. This rise correlates directly with the expansion of the center’s activities and growing public awareness. The leucistic bird described in this study was one of 48 Long-eared owls admitted during the 2016 calendar year.

**Figure 1:**
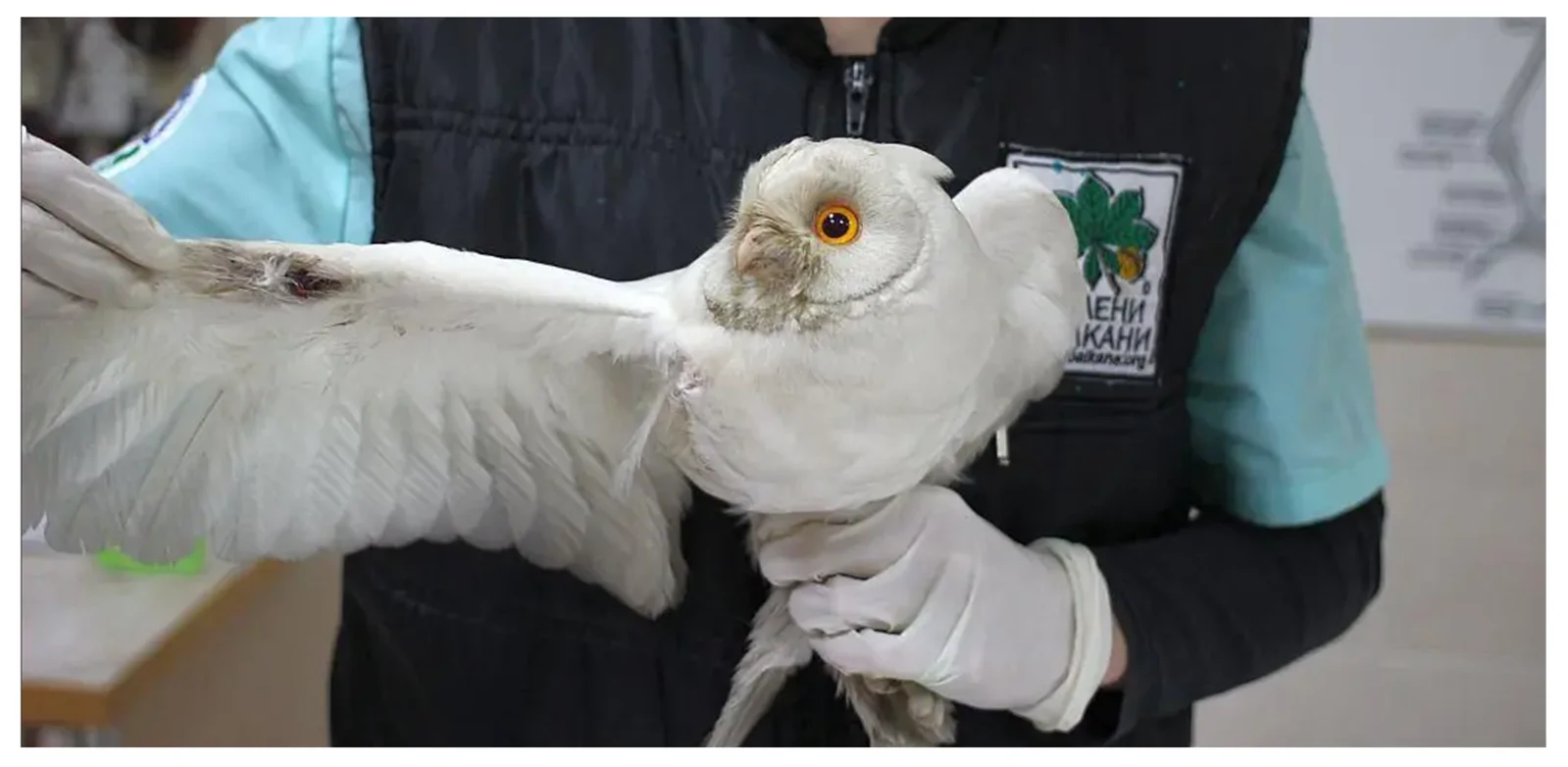
The leucistic Long-eared owl upon admission during clinical examination in the Wildlife Rehabilitation and Breeding Center. The image was taken under standardized light.

**Figure 2:**
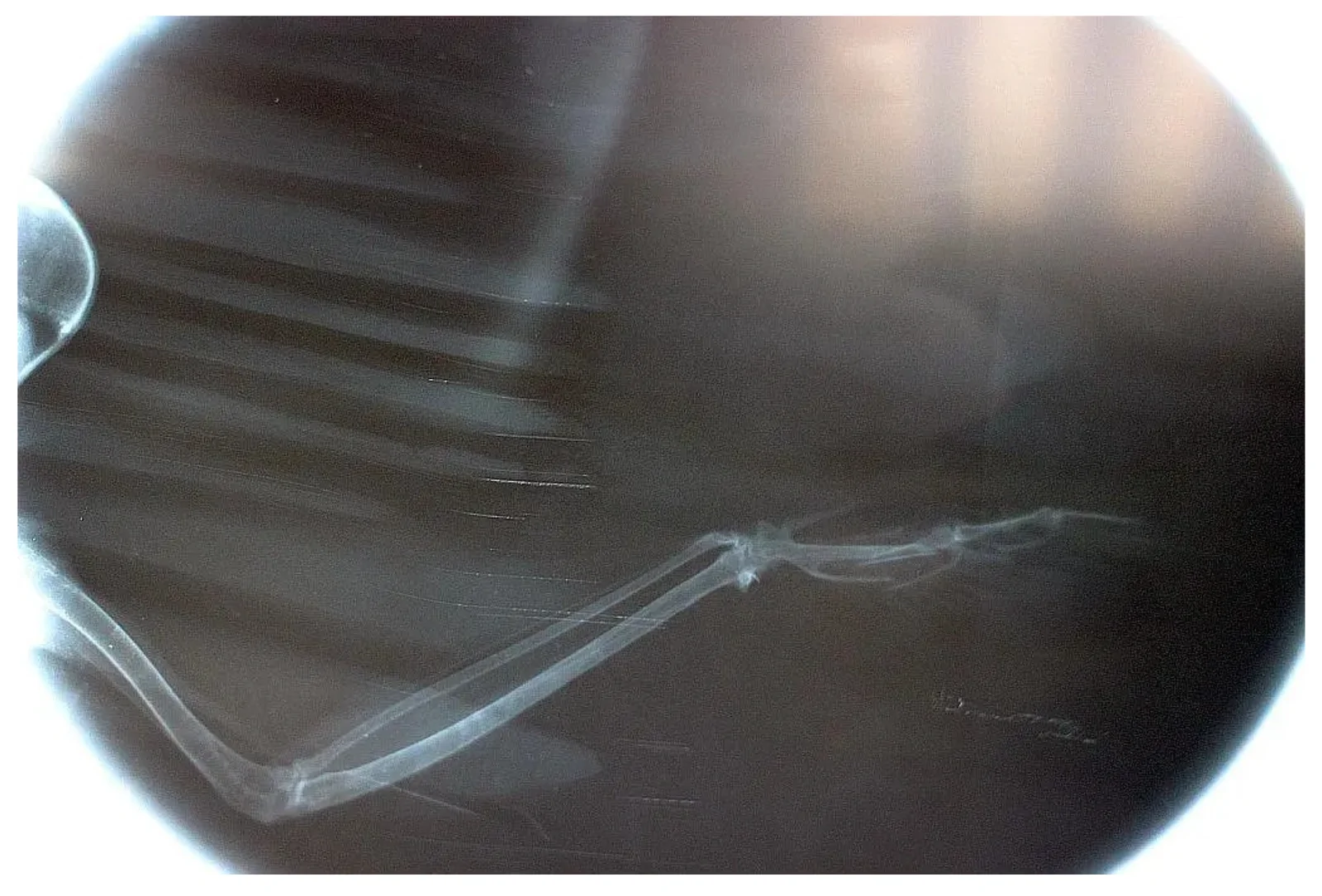
MedioLateral (ML) radiographic image of the Long-eared owl, showing multiple fractures of the metacarpal bones of the right wing prior to orthopedic stabilization.

The leucistic bird was admitted to the Wildlife Rehabilitation Center on 18 February 2016 (Case No. 0066/2016). The bird had been found in the village of Vasil Drumev, which is in the Shumen Province of Bulgaria, and was then sent to the center for treatment. Upon admission, the owl was in fair body condition but slightly thin, with a body mass of 240 g. The individual was formally identified using a metal ornithological ring from the Bulgarian Academy of Sciences (No. 5–105869). During its stay at the center, the bird was given the internal facility identifier “Nadejda” (Figure 1). Clinical examination and diagnostic radiography revealed a severe closed trauma featuring a multiple fracture of the metacarpal bones of the right wing (Figure 2). No blood examination or other invasive diagnostic assessments were performed.

**Figure 3:**
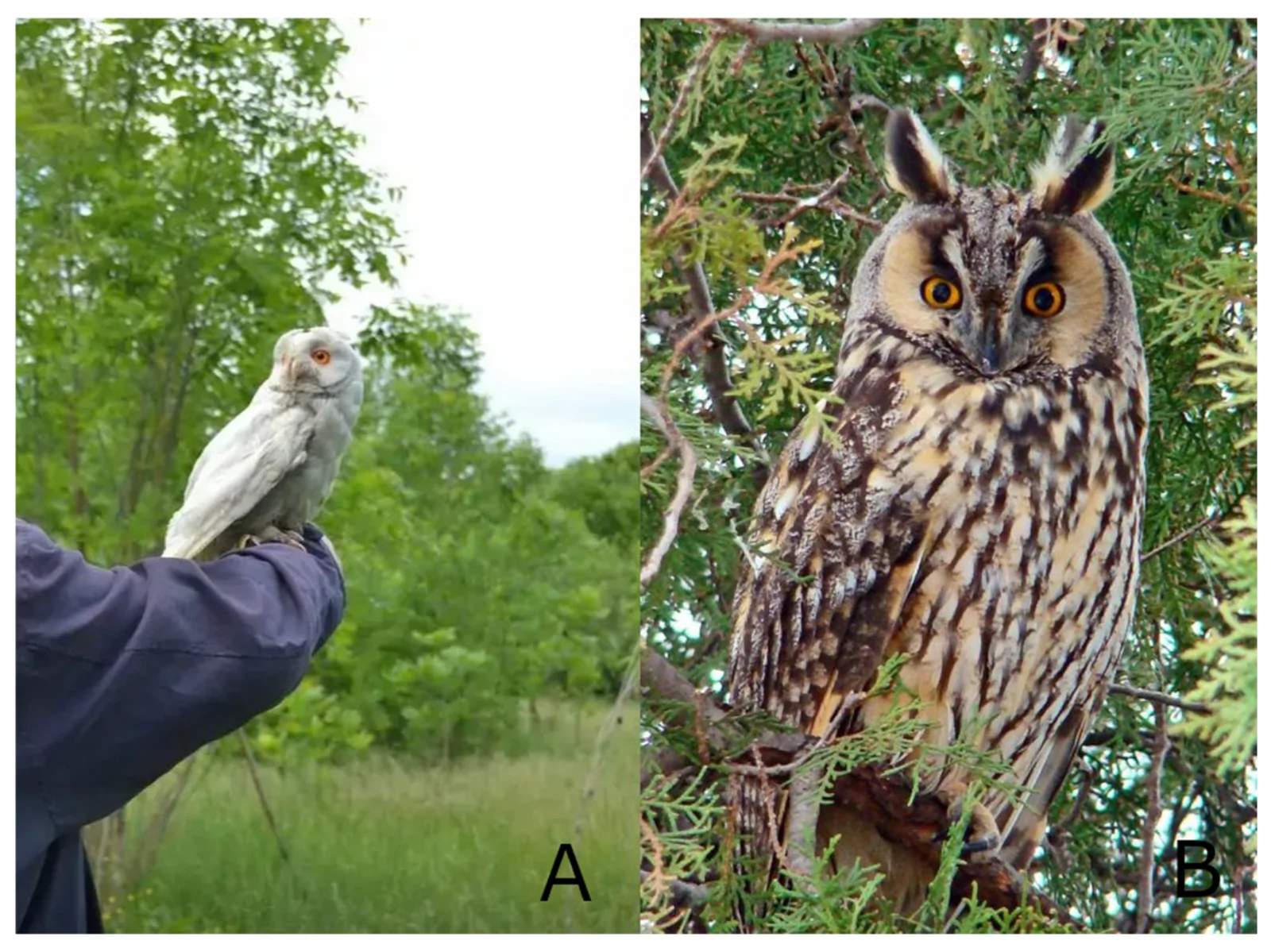
Phenotypic comparison of Long-eared owls: (**A**) Complete leucistic individual with all-white plumage, depigmented bare parts, and normal ocular melanin during the first release attempt (17 May 2016); (**B**) Typical wild-type individual exhibiting standard cryptic plumage with longitudinal and transverse dark stripes. Both photographs were taken under natural outdoor lighting.

The first clinical examination showed abnormal plumage coloring, consisting of a total lack of the species’ typical brownish-yellow color and instead featuring all snow-white plumage over the whole body (Figure 3A). The distal tissues, such as the rhamphotheca and podotheca, also showed complete depigmentation. The usual longitudinal and transverse dark stripes found in wild members (Figure 3B) of the same species were also missing. Even though the plumage was completely depigmented, the irides still had their normal orange-brown pigmentation and did not show the red or pinkish tint, which are found in albino types, where the tint appearance is caused by choroidal reflection, due to the total absence of pigment. Also, both the rhamphotheca and podotheca on the leucistic owl lacked normal integumentary pigmentation.

Despite the altered color of the plumage, all the main diagnostic morphological features are in accord with the species’ profile. Characteristics such as the distinct auricular tufts, the shape of the facial disc, the body proportions, and the structure of the plumage remain consistent with the species’ description. Apart from the pigmentary abnormality that has been observed, no other external morphological abnormalities were found.

To differentiate leucism from albinism and other hereditary pigmentary disorders, the phenotype was evaluated following the diagnostic criteria of van Grouw [4,6] and Hadad and Yosef [7]. The specimen was found to exhibit complete leucism. Evidenced by the total lack of pigmentation in the plumage, rhamphotheca, and podotheca, while the pigmentation of the irises remained standard.

To manage the severe wing injury, the owl underwent a conservative external immobilization without general anesthesia. The external immobilization was performed with an aluminum bandage, applied to the right wing for 21 days to facilitate bone healing, with joint mobilization, massage, and bandage replacement performed every 7 days. Systemic medical therapy was administered via intramuscular injection over a 7-day period, consisting of 0.15 mL daily lincomycin 50 mg–spectinomycin 100 mg/1 mL. (Alfasan^®^, Woerden, The Netherlands) for 7 days, 0.25 mL daily dexamethasone sodium phosphate 2,0 mg/1 mL (Alfadexx^®^, Woerden, The Netherlands) for 3 days, and a single 0.25 mL dose of vitamin B-complex (Alfasan^®^, The Netherlands). In total, the owl spent approximately four months (18 February to 24 June 2016) at the center undergoing veterinary treatment, physical rehabilitation, and flight training in a large enclosure (4 m × 4 m × 4 m). Fitness for release was confirmed following a comprehensive clinical evaluation by three veterinarians and flight performance testing. The first release attempt was made on 17 May 2016, in a park at the edge of Vasil Drumev village near the intake site. However, monitoring after the release revealed poor flight stability, expressed in short flights; landing on low vegetation or the ground, and insufficient altitude gain, which indicated that the wing required further muscle strengthening and flight recovery. As a consequence, it was sent back to the rescue center for further rehabilitation.

Second attempt to release the animal was made on 24 June 2016, near Yambol in a pine and fir park habitat, after it had been in recovery for an extended period and had been assessed for its flight ability. When it was released, the animal weighed 330 g, so there was a 90 g increase from its weight at admission. During the release, the owl demonstrated active flight, gained sufficient altitude, and perched securely on top of a pine tree approximately 7 m high, so it was successfully released. Following release, no post-release resightings, wild monitoring observations, or readmissions to the rescue center have been recorded.

Because plumage-based sexing criteria could not be applied due to the all-white plumage, sex was evaluated using body mass characteristics. Based on its body mass upon release (330 g), the individual was determined to be an adult female. Female Long-eared owls are significantly larger and heavier than males, which typically weigh under 280 g [8].

**Figure 4:**
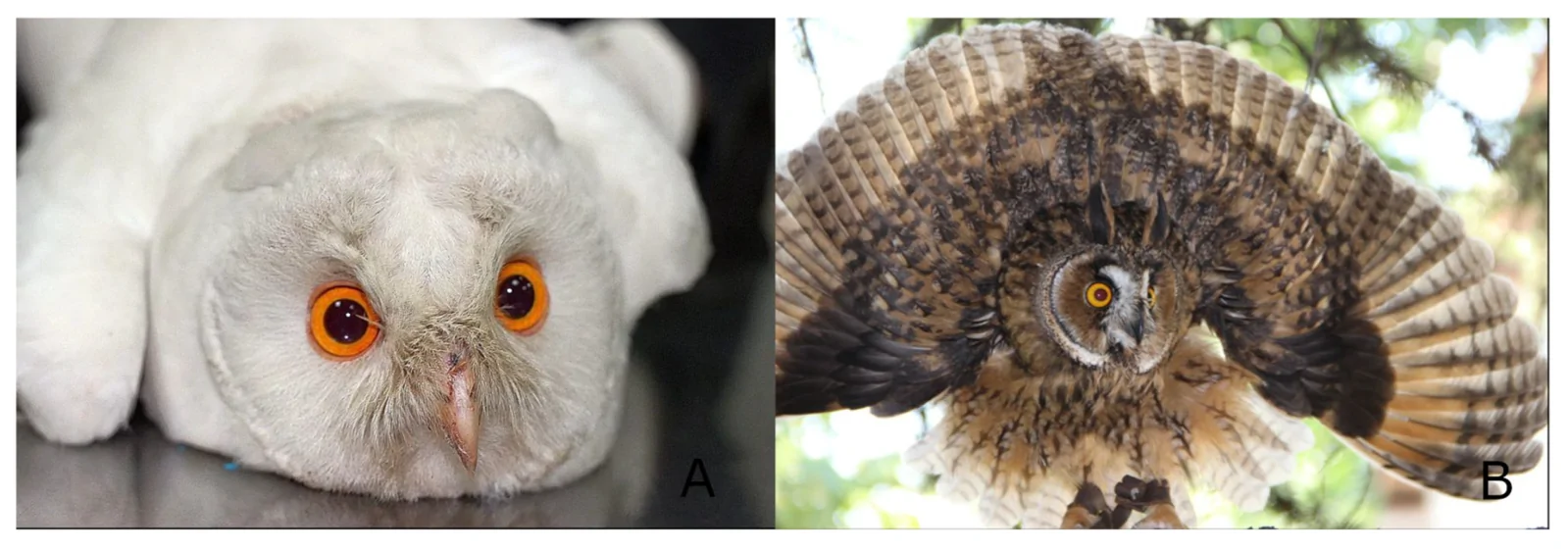
Facial detail comparison of Long-eared owls: (**A**) Leucistic individual showing normal orange ocular melanin upon admission and complete depigmentation of the facial feathers and beak (rhamphotheca), photographed under standardized indoor light; (**B**) Standard wild-type individual, photographed under natural outdoor light.

The photographic evidence taken at the time of admission shows the main characteristics of the pigmentary anomaly. These photographs give a full account of the leucistic specimen when it arrived at the rescue center, so they allow a comparison between a wild-type bird and the leucistic Long-eared owl to show the difference in plumage, and also that both irides retained normal orange-brown pigmentation without the pinkish or red choroidal reflection characteristic of albinism (Figure 4A), as compared to the head features of a normal wild-type individual (Figure 4B).

## 4. Discussion

In wild avian populations, pigmentary anomalies are infrequent phenotypic variations that could provide considerable insights into genetic diversity, mechanisms of plumage development, and the subsequent implications for individuals’ adaptive qualities [2,3]. Leucism is distinguished from albinism by the total or partial absence of melanin-producing cells in the plumage and skin, while ocular pigmentation remains unaffected [4]. Precise phenotypic differentiation between these conditions is of critical clinical and ornithological significance when documenting chromatic aberrations [6]. Although leucism frequently manifests as a partial condition affecting only scattered feathers, complete leucism involves the total absence of melanin across the entire plumage. If it extends to exposed integumentary structures such as the rhamphotheca and podotheca [4,6,15], then this manifestation is classified as a complete form of leucism rather than albinism, as ocular melanin remains present in the irides [16]. During the phenotype distinction, progressive greying was likewise ruled out, because the owl presented total depigmentation of both the plumage and bare parts immediately upon admission, rather than the patchy or variegated pattern, which is typical for progressive stages. Furthermore, during the 4-month rehabilitation period at the rescue center, no changes in plumage pattern or localized repigmentation were observed in regrowing feathers, leading to the idea that this is a completely stable phenotype. Other chromatic aberrations, such as dilution or ‘brown’, were excluded as they merely reduce melanin intensity—resulting in greyish, buff, or pale brownish plumage—whereas this specimen displayed pure snow-white feathers [4,6,7]. Similarly, ‘ino’ causes strongly bleached plumage accompanied by lightened, reddish irides [4,6]. ‘External staining’ was also excluded due to its patchy, locally concentrated discoloration characteristics [7]. Therefore, the combination of pure white plumage, depigmented bare parts, and normal ocular pigmentation tends towards the diagnosis of complete leucism [4,6,7]. In the absence of molecular or tissue-level verification, this classification remains strictly grounded in external phenotypic evaluation.

This study describes an instance of complete leucism in the Long-eared owl, a species in which this condition has not been previously recorded. According to an examination of the archival records kept by the Wildlife Rescue Center, out of the 1098 unique animals admitted between 1995 and 2026, only one leucistic owl was found (the admission frequency being 0.091%). This value represents the frequency of leucism in the center’s admission records and should not be interpreted as a population-level prevalence estimate, although national breeding monitoring [11] similarly recorded no such cases. These data confirm that the phenomenon is exceptionally rare. The results are consistent with findings from large-scale banding and intake studies on nocturnal raptors, which report a leucism frequency of less than 0.01% [17], as well as with individual records for other species within the same order—Strigiformes [14,18]. It is also notable that even within the rare pigmentary aberrations documented in large-scale studies, complete leucism represented only a fraction of all cases (a single partially leucistic individual among >30,000 birds) [17], further confirming the rarity of such color abnormalities in nocturnal raptors. It is well established that such recessive mutations in wild populations are often linked to high mutational load or inbreeding in isolated groups [19,20].

Documented instances of pigmentary abnormalities across the order Strigiformes remain scarce and vary significantly in their diagnostic reliability [7,14,21]. To evaluate these records accurately, it is important to distinguish opportunistic field observations from studies based on physical examination, museum specimens, or clinical admissions [6,8]. Several reported cases in owls rely primarily on brief field sightings or photographs, such as instances noted in the Burrowing owl (*Athene cunicularia*) [18], the American barn owl (*Tyto furcata*) [22], and the Magellanic horned owl (*Bubo magellanicus*) [23]. While valuable, distance observations rarely allow for the detailed inspection needed to evaluate soft-tissue depigmentation, confirm normal ocular melanin, or rule out progressive greying [4,6,7]. Conversely, records supported by physical handling, museum collections, or wildlife rescue center intakes—such as those reported for the Great horned owl (*Bubo virginianus*) [24], the Great gray owl (*Strix nebulosa*) [14], the Northern saw-whet owl (*Aegolius acadicus*) [17], and the Eurasian eagle-owl (*Bubo bubo*) and Little owl (*Athene noctua*) [8]—provide a higher level of diagnostic confidence. Since there are no previous records of the Long-eared owl, the current instance represents an important addition to the knowledge of chromatic aberrations in this species.

The full depigmentation seen in this individual could have serious ecological consequences. The loss of cryptic coloration reduces camouflage efficiency, potentially increasing conspicuousness to predators or prey [25]. Moreover, the decreased melanin level weakens the structural and mechanical strength of the feathers, which may make them far more prone to abrasion and premature wear [26]. Also, studies suggest that abnormally white individuals in wild populations may be exposed to a higher risk of predation and aggression from other species [20]. For nocturnal raptors, crypsis is important when they are roosting during the day. Conversely, to their benefit, is that specimens with normal iris pigmentation does not have higher light sensitivity nor they are prone depth perception impairment [4]. Also, white plumage may fail to trigger innate predator-recognition templates in prey, potentially delaying their flight response [27]. Crucially, while the bird recovered sufficiently to be released after veterinary treatment, successful rehabilitation does not demonstrate long-term wild survival, successful reproduction, or social integration. Complete leucism did not preclude short-term clinical recovery, body mass gain, or the restoration of active flight under captive conditions. However, because post-release systematic field monitoring was not performed, the owl’s survival and long-term fitness remain unknown.

Beyond genetic factors, an individual’s nutritional status and oxidative stress can also affect the quality of their feathers; the foods they eat provide the necessary precursors for normal metabolic processes. As a result deficiencies, could affect the structure and intensity of melanin-based pigmentation negatively. When being exposed to severe hunger, metabolic deficiency in Cu^2+^ can result in quantitative plasticity in coloration, even in the beak and tarsi [28]. Yet, the observation of complete depigmentation across both feathered and non-feathered integumentary structures cannot be an outcome of severe hunger. This argues against the idea that nutritional deficiency is the primary cause and instead points to a genetic mutation. It is more likely that nutritional status contributed significantly to tissue regeneration and physiological recovery during the rehabilitation process [29]. Moreover, unlike environmentally induced pigment suppression, which is transient and typically resolved in subsequent molt cycles following dietary restoration, true leucism represents a permanent, determined failure of melanocyte lineage maintenance that persists across all consecutive molts [4].

This case highlights both the scientific value and methodological limitations of wildlife rescue databases [30]. Rehabilitation centers provide multi-decade datasets of rare phenotypes, detailed clinical and photographic documentation, rehabilitation outcome records, and opportunities for future biological sampling (such as feathers or blood), for molecular or histological analyses [29,30]. However, rescue data do not reflect the entire wild population because people are much more likely to find and report birds near towns or accessible areas. Visually striking animals, as in this case, an all-white owl, are much easier for people to spot and report than normally camouflaged birds [30]. Conversely, such conspicuous aberrant birds could experience greater predation risk in the wild and may ultimately be underrepresented among live admissions. Furthermore, admitted animals represent an atypical group of injured or sick individuals rather than healthy wild populations. Therefore, birds admitted to a rehabilitation center cannot be considered directly representative of the broader regional population, and the admission frequency (0.091%) should not be interpreted as a true population-level prevalence. Understanding these limitations allows researchers to use the rescue center’s archives more effectively and accurately [30].

## 5. Conclusions

This report documents a rare phenotype consistent with complete leucism in a Long-eared owl admitted to a Bulgarian wildlife rehabilitation center. The occurrence of a single affected individual among 1098 admissions over a 31-year archival period highlights the immense value of long-term clinical databases for detecting uncommon chromatic abnormalities. Although the owl recovered and was successfully released into the wild, the ecological and fitness consequences of complete leucism in this species remain largely unknown. Future cases warrant further documentation, standardized photographic reporting, and, whenever feasible, genetic or histological investigation to confirm the underlying molecular mechanisms.

## Figures and Tables

**Table 1:** Admissions of Long-eared owls at the Wildlife Rescue Center grouped by 5-year intervals (1995–2026, *N* = 1098). Data for 2026 * cover the period up to 31 August.

Period	Number of Admitted Long-Eared Owls
1995–1999	8
2000–2004	75
2005–2009	250
2010–2014	274
2015–2019	237
2020–2024	215
2025–2026 *	39
Total:	1098

## Data Availability

The original contributions presented in this study are included in the article. Further inquiries can be directed to the corresponding author.
